# Putting together the pieces: building a reaction-centric electronic lab notebook for mobile devices

**DOI:** 10.1186/1758-2946-6-S1-O1

**Published:** 2014-03-11

**Authors:** Alex Clark

**Affiliations:** 1 1900 St. Jacques 302, Montreal H3J2S1, Canada

## 

The presentation will describe 4 years of work creating chemical structure based user interfaces for mobile devices, combined with creation of cloud-hosted webservices for supporting functionality.

A number of products have been created along the way, for drawing structures and reactions, managing collections of data, searching databases, creating publication quality graphics, sharing and collaborating, calculating properties, and model building for drug discovery research.

The focus will be on the assembly of these core technologies to create a fully featured electronic lab notebook (ELN) product for capturing chemical reactions.

While a number of web or mobile products already exist for laboratory notetaking, none of them provide the high level chemistry understanding and sophisticated cheminformatics functionality that will be described. The ability to combine all of the necessary capabilities onto a mobile device such as an iPhone or iPad demonstrates that this new generation of computing platforms is ready to play an important role in the realm of cheminformatics.

